# *“Pharmacies are Everywhere, and You can get it at any Time”*: Experiences With Pharmacy-Based PrEP Delivery Among Adolescent Girls and Young Women in Kisumu, Kenya

**DOI:** 10.1177/23259582231215882

**Published:** 2023-11-23

**Authors:** Melissa Vera, Elizabeth Bukusi, Pauline Achieng, Helen Aketch, Evelyne Araka, Jared M. Baeten, Kristin Beima-Sofie, Grace John-Stewart, Pamela K. Kohler, Melissa L. Mugambi, Bernard Nyerere, Josephine Odoyo, Caroline Omom, Christine Omondi, Katrina F. Ortblad, Jillian Pintye

**Affiliations:** 1 7284 School of Nursing and Department of Global Health, University of Washington, Seattle, WA, USA; 2 118982 Kenya Medical Research Institute, Kisumu, Kenya; 3 7286 Fred Hutchinson Cancer Research Center, Seattle, WA, USA; 4 Gilead Sciences, Foster City, CA, USA

**Keywords:** PrEP, adolescents, HIV prevention, pharmacy, contraception

## Abstract

**Introduction:**

Many Kenyan adolescent girls and young women (AGYW) with behaviors associated with HIV acquisition access contraception at retail pharmacies. Offering oral pre-exposure prophylaxis (PrEP) in pharmacies could help reach AGYW with PrEP services.

**Methods:**

We piloted PrEP delivery at 3 retail pharmacies in Kisumu, Kenya. AGYW purchasing contraception were offered PrEP by nurses with remote prescriber oversight. AGYW who accepted were provided with a free 1-month supply. We conducted in-depth interviews with AGYW 30 days postobtaining PrEP. Transcripts were analyzed to explore experiences of AGYW accessing PrEP at pharmacies.

**Results:**

We conducted 41 interviews. AGYW preferred pharmacies for accessing PrEP and they were willing to pay for PrEP even if available for free at clinics. Reasons for this preference included accessibility, lack of queues, and medication stockouts, privacy, anonymity, autonomy, and high-quality counseling from our study nurses.

**Conclusions:**

Pharmacies may be an important PrEP access option for this population.

## Introduction

Adolescent girls and young women (AGYW) in Kenya experience persistently high HIV incidence.^1^ Despite ongoing efforts to increase pre-exposure prophylaxis (PrEP) access among AGYW in Kenya, initiation in this priority population remains low, with evidence suggesting that only 4% to 16% of Kenyan AGYW initiate PrEP when offered in family planning (FP) clinics.^2–4^ AGYW seeking sexual and reproductive health services face unique challenges, such as stigmatization over behaviors associated with HIV acquisition, decreased ability to access healthcare due to transportation and school/work schedules, and a dissuading fear of stigma related to seeking and obtaining PrEP.^5–10^ To maximize the HIV prevention impact of PrEP for AGYW, there is a need for wider availability of PrEP access points, especially for AGYW who do not frequently seek care at health clinics. Retail pharmacies are utilized for many reasons and provide relative anonymity and stigma reduction to those who purchase PrEP. Recent research demonstrates that retail pharmacy-based PrEP delivery is acceptable and preferred in non-AGYW populations.^6,11–14^ To date, few data on pharmacy-based PrEP delivery exist among AGYW but available evidence suggests feasibility.^4^

Our team recently completed a pilot evaluation of nurse-facilitated PrEP delivery at retail pharmacies in Kisumu, Kenya.^4^ We found that 85% of AGYW participants seeking contraception at retail pharmacies accepted daily oral PrEP when offered and 69% of those who initiated PrEP were willing to purchase it at retail pharmacies even if it is available for free in public clinics. To complement our quantitative results, we conducted a qualitative evaluation to understand AGYW experiences with pharmacy-based PrEP, including reasons for preferring pharmacy-based PrEP delivery as this is not well characterized among AGYW to date. Our overall objective was to inform implementation of pharmacy-based PrEP for AGYW in Kenya by understanding and describing experiences with pharmacy-based PrEP among AGYW.

## Methods

### Study Design and Population

This qualitative study was nested in a pilot evaluation of pharmacy-based PrEP delivery among AGYW seeking contraception at 3 retail pharmacies in Kisumu, Kenya. Recruitment, eligibility and exclusion criteria, follow-up procedures, and detailed study participant characteristics have been previously described.^15^ Briefly, from October 2020 to March 2021, pharmacy personnel referred female clients aged 15 to 24 years seeking contraception to our study nurses who then screened them for eligibility, continuing to enroll eligible AGYW until 200 participants accepted PrEP pills. All AGYW in this study self-identified as cisgender women. We used nurses in this study for their communication skills and experience with counseling AGYW in HIV prevention and other topics of health, aiming to create a space where AGYW could receive ample one-on-one time with a practitioner for education regarding PrEP initiation, which is not always possible in the pharmacy setting. AGYW aged 15 to 24 years who purchased contraception (emergency contraception [EC], oral contraceptive pills, injectables, implants, condoms) at the retail pharmacy and were willing to discuss PrEP were eligible for the pilot study. Study nurses provided counseling that followed Kenyan national guidelines, performed HIV tests on AGYW using OraQuick kits, provided OraQuick HIV self-test kits to AGYW to take home for partner testing, and PrEP pills to all eligible AGYW at the participating pharmacies under supervision of a remote physician. Additionally, nurses counseled on topics raised by AGYW, including contraceptive options and issues related to relationships and navigating PrEP use.

At 30 days postobtaining of PrEP pills, AGYW were purposively recruited in-person or via telephone for in-depth interviews (IDIs) from the 200 AGYW who accepted PrEP pills in the pilot study and currently had a male sexual partner. IDIs were intended to capture individual experiences with pharmacy-based PrEP delivery. The purposive sampling strategy aimed to have a balance of AGYW who did and did not initiate PrEP use after accepting PrEP pills to glean more information on the reasons AGYW decide to take PrEP or not. Those who met IDI eligibility criteria were offered participation in the IDIs by the study nurse until 41 AGYWs were recruited. All IDI participants completed interviews.

### Data Collection

Following IDI informed consent and enrollment, trained qualitative interviewers (HA and CO) recorded basic demographic information of each participant (age, gender, and relationship status). Semistructured IDI guides were created collaboratively between the study team members (JP, HA, CO, EB, and JO) and were based on literature reviews and research experience in this population and location. IDI guides were piloted prior to implementation by field staff and covered themes related to family planning, HIV prevention, and pharmacy services. IDIs were conducted by experienced interviewers (HA and CO) who identify as young Kenyan women. IDIs took place in either a private room within the retail pharmacy or at the Kisumu research study office, depending on space availability and participant preferences. IDIs were conducted in Kiswahili, Dholuo, or English based on participant preferences. Interviews took 45 min on average and were audio recorded and transcribed verbatim into English by interviewers (HA and CO). Initial transcripts were independently verified against the original audio file by the primary investigator (MV), who was a female-identifying PhD candidate. Repeat interviews and review of transcripts for correction were not offered to participants due to COVID-19-related travel restrictions and logistical challenges.

### Data Analysis

Using the principles of thematic analysis,^16,17^ the research team reviewed transcripts to code, identify, and evaluate themes related to positive user experience concepts from the High-Quality Health System Framework. This framework came from a need to redefine what defines high-quality health systems, their processes, inputs, and concepts. This framework was selected due to the study's evaluation of positive and/or negative experiences using pharmacies for PrEP delivery. Our study focused on 2 concepts under positive user experiences: respect (dignity, privacy, nondiscrimination, autonomy, confidentiality, and clear communication) and user focus (choice of provider, short wait times, patient voice and values, affordability, and ease of use).^18^ Transcripts were uploaded into Dedoose version 9.0.54 (SocioCultural Research Consultants, LLC, Los Angeles, CA, USA). A coding team, comprising the primary investigator (MV) and 2 additional study staff from Kenya (HA and CO), developed a codebook from a close read of a subset of 10 transcripts using inductive and deductive methods. Our team hypothesized that we would receive responses about the quality of health systems, therefore deductive codes were derived from concepts of user experience in the High-Quality Health System Framework. Inductive codes were created during multiple reviews of the transcripts by the coding team that revealed additional concepts not represented in the High-Quality Health System Framework, such as relationship dynamics. The codebook was developed as an iterative process, with the refinement of codes and code definitions occurring through repeated discussion and consensus as additional transcripts were reviewed. All transcripts were coded independently by a member of the coding team (HA, CO, and MV) using a final version of the codebook, and received a secondary review by another coding team member (HA, CO, and MV). Disagreements in coding were resolved through discussion. Coded data were summarized in visual matrices to identify themes across all IDI transcripts.

### Ethical Considerations

This study involved field procedures in Kisumu, Kenya and data analyses in Kisumu and Seattle, Washington. The Kenya Medical Research Institute Scientific and Ethics Research Committee (KEMRI/SERU/CMR/P00127/3956) and University of Washington Human Subjects Review Committee (STUDY00008868) reviewed and approved the study protocol, informed consent forms, and data collection tools. All study participants received a KES 300 (approximately USD 3) reimbursement for their time at each study visit and were welcome to end the study visit and/or participation at any point.

## Results

Overall, 41 AGYW participated in IDIs. The median age for all participants was 18 years (interquartile range [IQR] 16-20.25) and their partners’ median age was 25.8 years (IQR 22-29). The most frequently purchased contraceptive method at enrollment into the parent study was EC (49%, n = 20) followed by condoms (27%, n = 11). Among all participants, 49% (n = 20) accessed EC more than twice in the past 6 months. Almost two-thirds (63%, n = 26) of participants did not know their partner's HIV status and 100% (n = 41) had sex without a condom in the past 6 months. Of the 41 sampled participants, 22 (54%) started taking PrEP after acceptance and 19 (46%) did not start taking PrEP after initially accepting it in the pharmacy (Table 1). Reasons for not initiating PrEP included no longer feeling at risk for HIV acquisition, not having sex within the last month, and male partners testing HIV negative with the self-test kits participants took home. Some participants had not yet initiated PrEP use but planned to do so in the future when they would be sexually active (eg, boyfriend returns, school break, etc).

**Table 1.** Demographic Characteristics of Participants (N = 41).

Variable	N (%) or mean (IQR)
Pharmacy site	
Lolwe	20 (49%)
Medstar	16 (39%)
Win-Win	5 (12%)
Age (years)	18.2 (16-20.25)
Age of partner (years)	25.8 (22-29)
Education level In college Secondary school Vocational/trade None	9 (22%) 9 (22%) 2 (5%) 21 (51%)
Years of completed education	11.3 (10-12)
Relationship status Casual partners only One primary + casual partners One primary only Single	3 (7%) 11 (27%) 26 (63%) 1 (2%)
Partner provides financial support Yes No	35 (85%) 6 (15%)
Primary sexual partner has other partners No other partners Unsure Partner has other partners	13 (32%) 19 (46%) 9 (22%)
Partner HIV status Negative Unknown	15 (37%) 26 (63%)
Employment status Unemployed Currently employed	38 (93%) 3 (7%)
Ever had HIV test before enrollment visit Yes No	39 (95%) 2 (5%)
Condomless sex* Yes No	41 (100%) 0 (0%)
Exchanged sex for money or favors* Yes No	8 (20%) 33 (80%)
Consumed alcohol in past 30 days Yes No	14 (34%) 27 (66%)
Used EC more than twice* Yes No	20 (49%) 21 (51%)
Experienced forced sex Yes No	2 (5%) 39 (95%)
Experienced intimate partner violence Yes No	2 (5%) 39 (95%)
Ever had a previous pregnancy before Yes No	16 (39%) 25 (61%)
Ever heard of PrEP before enrollment visit Yes No	33 (80%) 8 (20%)
High self-perceived HIV risk Yes No	35 (85%) 6 (15%)

*In the past 6 months.

Abbreviations: EC, emergency contraception; IQR, interquartile range; PrEP, pre-exposure prophylaxis.

Three key themes surfaced from the IDIs about positive user experiences with pharmacy-based PrEP delivery, all relating to the *processes of care* domain in the High-Quality Health System Framework: (1) pharmacies are more accessible than health facilities, (2) client-centered communication increases interest in PrEP, and (3) pharmacies provide anonymity and decrease stigma. Further, HIV self-tests complemented user experiences with PrEP delivery due to ease of use, painless and efficient testing process, privacy, and peace of mind provided by knowing one's HIV status or their partner's status.

### Pharmacies are More Accessible Than Health Facilities

Participants consistently reported that the pharmacies were preferred for obtaining PrEP compared to health facilities because of easy access. Many participants reported not having reliable transportation to health facilities which may be located outside of hubs. Additionally, participants reported that PrEP is not universally or reliably available at all facilities in the community which makes it challenging to locate. Pharmacies were considered a better option because of their extensive locations across communities. Many AGYW walk from place to place, making pharmacies an attractive alternative for accessing both FP and HIV prevention care.

“*The pharmacy is easily accessible. If I want PrEP, I will not run to the hospital when the pharmacy is next door. I will go to the pharmacy.” (age 22, initiated PrEP)*

“*It is so easily accessible since pharmacies are everywhere and you can get it at any time and any point, unlike the hospital.” (age 22, did not initiate PrEP)*

Participants also reported retail pharmacies have longer hours of operation than hospitals and clinics, which offers more opportunities to access PrEP given typical work and school schedule restraints. The burden of getting time off work or to take time out of class were reasons given by participants for not going to a healthcare facility for PrEP.

“*Maybe, one can come from a far place and if it is not [open at] night then you can be stressed on how to get PrEP. But when it's [open at] night, you can just come at any time.” (age 20, did not initiate PrEP)*

Some participants stated that hospitals are limited in the days they offer certain services or medications. This was seen as a barrier by some participants. If a person wants to initiate PrEP use or obtain a refill, not only would they have to potentially take time off from work or school and get transportation to a healthcare facility, but they may also be turned away because it was the wrong day for PrEP distribution.

“*At the hospital, there are limits. You will go on a certain day, and they tell you that today they are not issuing [PrEP]. It is not every day that [PrEP is] issued at the hospital pharmacies. They have specific days for [PrEP]. So, you know with a [retail] pharmacy, at any time you want, you can easily access it.” (age 22, did not initiate PrEP)*

Many participants reported that facilities frequently have long wait lines, which was a deterrent and significant barrier to accessing PrEP at facilities. One participant reported waiting in line for over 2h for PrEP in a crowded waiting room. This was seen by the participant as a “waste of time” and an unwanted chance to experience stigmatization by others waiting in the queue, which was a reported consideration when deciding whether to continue taking PrEP. Participants perceived pharmacies as a better “value for the time” option for accessing PrEP, especially for AGYW who tend to have less acute reasons to attend healthcare facilities (eg, seeking contraception or preventive services) and are therefore treated with less urgency in those settings.

“*In the hospitals, you may find a lot of people in the line. You know the pharmacy is quick. At the hospital, maybe there is a queue. You fear the queue.” (age 22, initiated PrEP)*

“*Since you have come there [the health facility] for the drug [PrEP] and they know you are not that sick, they tend to ignore or just let young women sit there. While at the pharmacy…you can just go and be given what you want.” (age 20, did not initiate PrEP)*

Among IDI participants, over half (54%) reported they were willing to pay for PrEP even if it was freely offered in clinics. Preferring to pay at pharmacies over getting PrEP for free at clinics reported by AGYW include the convenience of pharmacy locations, the lack of queues and medication stockouts, and the lack of stigma at retail pharmacies when making purchases.

“*I heard that at the pharmacy, they sell [PrEP], and the hospital is where you are given [PrEP] for free. But, you know, in the hospital it is very hard to go and get [PrEP].” (age 22, did not initiate PrEP)*

“*For example, you’ve ran out of drugs, and you went to the hospital and find the long queue…and when you get to the counter they have run out of stock. It will force you to go the following day so it will be like you will be missing your drug for the day.” (age 17, initiated PrEP)*

*“Personally, the pharmacy is a good venue for me because they are quick. If you go to the hospital to access PrEP, there is no PrEP. If you come on that day, they tell you to come another day and if you go again, you don’t find it. So, to me pharmacy is good because here if you come…they just give it to you, but [hospitals] they tell you to come another day…” (age 18, initiated PrEP)*

### Client-Centered Communication Increases Interest in PrEP

Client-centered communication was a crucial factor in initiating PrEP in the retail pharmacy. Some participants reported that the study nurse had a “good attitude,” which made it easier to talk about health issues. Behaviors that demonstrated having a “good attitude” were eye contact, listening to the participant, asking questions and leaving time for the participant to ask questions, showing genuine care for the participant's needs and values, and being kind and approachable in demeanor. This provided a perceived safe space for participants, which encouraged PrEP initiation.

“*You know when you go to the hospital, maybe that person [PrEP provider] gives you an attitude that you can’t even open up to that person. But with the pharmacy, personally, I felt very comfortable. So, attitude makes people prefer pharmacy to the hospital.” (age 22, initiated PrEP)*

“*I came to buy a medicine and [the study nurse] welcomed me really well. She told me that she wants to talk to me about something that will help me. So, we talked and talked and she explained so many things and I thought it was a good idea.” (age 16, initiated PrEP)*

Providing adequate information regarding medications in general was perceived by participants to be an important aspect of client-centered communication. Participants cited both contraception and PrEP education from the study nurse as highly satisfactory with little room for confusion or unanswered questions regarding both medications. Some participants felt that the lack of information provided in public hospitals was a reason they preferred the retail pharmacy.

“*At the hospital, you will just go, and request contraception and they will give you without informing you more about it. You will not be told about the side effects. But [at the pharmacy], you can get time, ask the nurse how it works, any side effects. You can get to know more.” (age 23, did not initiate PrEP)*

In the retail pharmacy, participants were allowed time to get to know more about PrEP, which built safety and trust among the participants. The study nurse was seen as a trusted source to guide medication decisions because she built rapport and gave well-rounded education regarding PrEP.

“*The [study nurse] created an environment where we could share anything with her and feel safe. She was also so informative. She clearly told me everything I needed to know about PrEP.” (age 22, did not initiate PrEP)*

More quality time was reported by almost all participants to be of utmost importance when deciding where to go for medication needs. PrEP was perceived by participants as a medication that required time for explanation. Some participants voiced concerns over not knowing how to take the medication and what side effects to be aware of. These participants stated that they would prefer the retail pharmacy over healthcare facilities because they had time to speak with the study nurse, which is not the case in healthcare facilities.

“*At the pharmacy, I am not hurried. I take my time and there is a nurse who will be patient with me. But at the hospital, people are many and they rush you so you will not get enough time to be open and talk to a nurse for long.” (age 23, did not initiate PrEP)*

Some participants felt that the study nurse taking the time to provide in-depth education was the deciding factor to initiate PrEP.

“*Then [the study nurse] asked me if I would mind to learn more about PrEP. Then I said ‘yes’ because I was curious to know this PrEP. Yes, then she explained to me everything, that is when I made my decision [to accept PrEP pills].” (age 22, did not initiate PrEP)*

### Pharmacies Provide Anonymity and Decrease Stigma

Almost all participants reported that privacy was of great concern for them regarding PrEP access. The retail pharmacy was an ideal option because one could be going to the pharmacy for a multitude of reasons and not be stigmatized for overtly purchasing PrEP. One participant was specifically drawn to the retail pharmacy due to its relatively stigma-free space.

“*My main like was that, at the pharmacy, you know it is a place where you come and go and unlike, let's say a hospital, where you frequently visit when you are sick. [You can] come get your PrEP without stigmatization or someone judging you as a sex worker or something. The fact that you won’t get stigmatized is what really caught my attention.” (age 16, initiated PrEP)*

Many participants perceived utilizing hospitals as a signal to others that they are ill. The overarching fear reported is that people in the community will think they are living with HIV and discriminate against them due to that perception. Participants desired avoidance of HIV-related stigma. Many participants specifically stated they did not want people in their community to think they were taking antiretroviral therapy (ARVs) for treating HIV.

“*You have come [to a health facility], and you meet one of your neighbors there. It is always one room where people access this PrEP and also [ARVs]. You have come for PrEP, but later you hear them say ‘so and so is also taking this drug of ours [ARVs], we were with her at the hospital.’ This discourages me. You say to yourself, ‘So how will I go? People will think that I also take ARVs,’ but for me I know I have just gone for a drug that is going to protect me.” (age 23, initiated PrEP)*

Some participants felt that healthcare facilities had too many people waiting and talking, even the healthcare workers. This situation made some participants feel like their privacy was not assured when community members were waiting for care and the healthcare workers were talking among themselves.

“*In hospitals there are so many people. They have a bench and people are seated all over…people looking at you so I even fear explaining that this is what brought me [in]. Then, you know, public hospitals [are] noisy. Nurses shout, make noise, discuss stories, and laugh even if you are there…so they even make it difficult for me to share my issues…when they come in and laugh, you feel guilty. But here at the pharmacy, it is peaceful, you can open up and talk about your personal issue and get help.” (age 23, initiated PrEP)*

Another aspect of privacy and retail pharmacies that was mentioned among participants is the control of participants’ time while in the pharmacy. At a clinic or hospital, a patient is called when it's their turn, regardless of what's going on around them in the greater environment. Some participants reported feeling safer being able to control when they go into a pharmacy, or even when to go up to the pharmacist to purchase PrEP. They could wait for a time when they felt comfortable.

“*That is the same way people do with [emergency contraception]. You wait, even if they have many customers, you stand at a distance and wait. When they are all gone, you run, purchase the drug and leave. You can even ask the pharmacist to wrap it in a way that somebody won’t know what you are carrying.” (age 22, initiated PrEP)*

### HIV Self-Tests Complement Pharmacy-Based PrEP Delivery

HIV self-tests for partners were liked by most participants and complemented the experience of pharmacy-based PrEP delivery by amplifying another important facet to HIV prevention care: partner and self-knowledge of HIV status. Reasons for the positive regard for self-tests were that they were free, easy to use, quick, painless, provided peace of mind, a sense of safety because of status knowledge, and it could be done in the privacy of their home with or without their partner.

“*The positive effect is that I got peace of mind. I also got my sex partner's HIV status according to the self-test I was given. I now knew his status, so even if we were having sex, I knew that I was safe.” (age 23, did initiate PrEP)*

“*What I liked was that [self-testing at home] was between us, we had privacy; we are in the house testing together and I liked it. What I also like is that no one forced the other. We were both willing and there was no forcing*.” *(age 23, did initiate PrEP)*

The only negative comments were related to cost and being alone with a self-test kit. A few participants thought being alone taking a self-test might become a problem if that was how someone found out they had tested positive for HIV.

## Discussion

This qualitative evaluation is part of a pilot study exploring the acceptability and user experiences of AGYW accessing PrEP in the pharmacy setting in Kisumu, Kenya. The quantitative^15^ evaluation found that the majority of AGYW in the pharmacy prefer obtaining PrEP from retail pharmacies over health facilities. Through IDIs, we found that the reasons for this preference include greater accessibility, superior client-centered communication from study nurses, and the relative anonymity provided in the retail pharmacy setting. More specifically, participants decided to accept PrEP pills based on the quality of care and privacy provided in the retail pharmacy setting. These findings contribute to prior quantitative studies^3,4,6^ by explaining and identifying reasons for the overall preference of pharmacy-based PrEP delivery over health facility-based models. Our findings highlight concerns AGYW have when seeking PrEP and the potential for pharmacy-based PrEP delivery to address these concerns and maximize PrEP access for AGYW in Kenya.

Our findings suggest that addressing concerns beyond PrEP (ie, contraceptive methods) in tandem with HIV prevention may be an effective approach to responding holistically to sexual and reproductive health needs of AGYW. Nurses addressed other concerns during counseling (eg, relationship issues, contraception, and HIV testing) which was valued by AGYW, though this approach may not be scalable outside of research settings. We are testing the cost-effectiveness of utilizing nurse-navigators for AGYW within a pharmacy-based PrEP delivery model.^19^ In a 2021 study, Donnell et al^20^ found a significant decrease in HIV acquisition among participants accessing contraception with PrEP offer (2.2 HIV seroconversions in 100 person-years) compared to those accessing contraception when PrEP was not available on-site (4.6 HIV seroconversions in 100 person-years). High frequency of having condomless sex, partners of unknown HIV status, and high self-perceived HIV risk among participants in our pilot suggests AGYW may especially benefit from PrEP delivery when integrated where they already access contraception.

Our study found that client-centered communication is essential for high-quality interactions between AGYW and study nurses in pharmacies. Client-centered communication incorporates using open-ended questions early on to ascertain the client's values and health goals, not interrupting, using active listening techniques; all which convey understanding, support, care, and empathy toward the client being served.^21^ These communications skills were used by our nurses and are a tangible approach for any PrEP provider to ascertain health concerns of AGYW clients to offer the most appropriate services possible in one place.^22^ AGYW with behaviors associated with HIV acquisition may be especially vulnerable to stigma while also being those most likely to benefit from PrEP. Similarly, supportive provider communication facilitated adolescent HIV testing^23^ and AGYW contraception services.^24^ Well-trained, client-centered communicators at PrEP access points could promote PrEP initiation among AGYW by addressing their unique needs and questions.^25^

Accessibility was a key theme in our study. Generally speaking, access to retail pharmacies improves health outcomes by providing a conduit for life-saving tests and medications beyond PrEP and contraception. Maloney et al^26^ found that providing access to malarial testing in retail pharmacies in Tanzania increased malaria diagnoses and treatment of malaria infection. Rutta et al^27^ found that retail pharmacists in Tanzania are key in aiding tuberculosis detection and treatment. In a 2016 study,^28^ an increase in adult vaccinations followed an increase in the availability of vaccines at retail pharmacies, decreasing vaccine-preventable deaths. This evidence laid the groundwork for the COVID-19 vaccine response, where retail pharmacies played a huge role in increasing vaccination rates.^29^ In the context of AGYW and HIV prevention, pharmacies could play a broad role in improving health outcomes by providing OraQuick HIV tests, eligibility screenings for PrEP, and wide access to PrEP medication.

Privacy in PrEP delivery was a core concern among AGYW in our study. Privacy provides a safe space for people to receive care where otherwise they would not. Studies on opioid dependence in Australia^30^ and sexual health in Britain^31^ both found that private spaces where one can speak openly to a health provider in the pharmacy setting was a key factor among marginalized populations and those with sensitive health concerns seeking and maintaining care. AGYW are particularly vulnerable to stigma and need more access points for testing and medication purchasing options. Retail pharmacies could play a pivotal role in providing lasting care due to the access and privacy they provide.

### Limitations

Our study uses data from only 3 retail pharmacies and may not be generalizable to the greater communities of Kenya or other HIV high-burden countries. We sampled AGYW already in the retail pharmacy setting accessing contraceptives, which likely does not include AGYW who experience further barriers to pharmacy-based PrEP initiation. Our sample also does not include AGYW who prefer FP and Maternal-Child Health (MCH) clinics for PrEP access, thus leading to a potential bias in reported preference of pharmacy over clinic. These AGYW who were not recruited may have more intense or different challenges that we are unable to include in these analyses. Most AGYW in our sample had high self-perceived HIV risk, which is associated with PrEP use in other settings, and therefore willingness to start PrEP may not be representative of all AGYW in this setting. We were not able to offer follow-up interviews with participants for transcript verification due to budget constraints and the COVID-19 pandemic. Further, the COVID-19 pandemic restrictions could have had an effect on AGYW willingness to seek PrEP in a pharmacy versus a clinic. Lastly, our study nurses may differ from other nurses in public health spaces, providing a skewed perspective of how a nurse in a retail pharmacy would be perceived by AGYW.

## Conclusions

AGYW seeking contraception is a critically important population to reach with HIV prevention services. It is crucial to expand PrEP delivery access point to reach a larger proportion of the AGYW population, especially those that may not frequently access health facilities. Future research is needed that includes a broader sample of pharmacies and AGYW participants to further evaluate pharmacy-based PrEP in this population. With the emergence of novel PrEP agents (eg, vaginal ring, injectables, etc), further research is needed that incorporates these new models within a variety of delivery platforms, including further research into the scale-up and sustainability of nurse-led PrEP delivery models.

## References

[1] Carter A Msemburi W Fraser MS , et al. Estimates of global, regional, and national incidence, prevalence, and mortality of HIV, 1980–2015: the Global Burden of Disease Study 2015. Lancet (British Edition). 2016;3(8):e361–e387. doi:10.1016/S2352-3018(16)30087-X PMC5056319 27470028

[2] Mugwanya KK Pintye J Kinuthia J , et al. Integrating preexposure prophylaxis delivery in routine family planning clinics: a feasibility programmatic evaluation in Kenya. PLoS Med. 2019;16(9):e1002885. doi:10.1371/journal.pmed.1002885 PMC6719826 31479452

[3] Pintye JJ Kinuthia JJ Roberts DADA , et al. Integration of PrEP services into routine antenatal and postnatal care: experiences from an implementation program in Western Kenya. J Acquir Immune Defic Syndr. (1999). 2018;79(5):590-595. doi:10.1097/QAI.0000000000001850 PMC6500722 30204720

[4] Pintye J Odoyo J Nyerere B , et al. Nurse-facilitated PrEP delivery for adolescent girls and young women seeking contraception at retail pharmacies in Kisumu, Kenya. AIDS (London). 2022;37(4):617-623. doi:10.1097/QAD.0000000000003447 PMC9974532 36653342

[5] Kennedy CE Yeh PT Atkins K Ferguson L Baggaley R Narasimhan M . PrEP distribution in pharmacies: a systematic review. BMJ Open. 2022;12(2):e054121. doi:10.1136/bmjopen-2021-054121 PMC8860049 35190430

[6] Begnel ER Escudero J Mugambi M , et al. High pre-exposure prophylaxis awareness and willingness to pay for pre-exposure prophylaxis among young adults in Western Kenya: results from a population-based survey. Int J STD AIDS. 2020;31(5):454-459. doi:10.1177/0956462420912141 32208816 PMC7291793

[7] Holzemer WL Uys L Makoae L , et al. A conceptual model of HIV/AIDS stigma from five African countries. J Adv Nurs. 2007;58(6):541-551. doi:10.1111/j.1365-2648.2007.04244.x 17484748

[8] Giovenco D Gill K Fynn L , et al. Experiences of oral pre-exposure prophylaxis (PrEP) use disclosure among South African adolescent girls and young women and its perceived impact on adherence. PLoS One. 2021;16(3):e0248307. doi:10.1371/journal.pone.0248307 PMC7935254 33667264

[9] Jani N Mathur S Kahabuka C Makyao N Pilgrim N . Relationship dynamics and anticipated stigma: key considerations for PrEP use among Tanzanian adolescent girls and young women and male partners. PLoS One. 2021;16(2):e0246717. doi:10.1371/journal.pone.0246717 PMC7888654 33596216

[10] Velloza J Khoza N Scorgie F , et al. The influence of HIV-related stigma on PrEP disclosure and adherence among adolescent girls and young women in HPTN 082: a qualitative study. J Int AIDS Soc. 2020;23(3):e25463. doi:10.1002/jia2.25463 PMC7060297 32144874

[11] Ortblad KF Mogere P Bukusi E Ngure K Baeten JM . Pharmacy delivery to expand the reach of PrEP in Africa. J Int AIDS Soc. 2020;23(9):e25619. doi:10.1002/jia2.25619 PMC7525802 32996721

[12] Dietrich JJ Atujuna M Tshabalala G , et al. A qualitative study to identify critical attributes and attribute-levels for a discrete choice experiment on oral pre-exposure prophylaxis (PrEP) delivery among young people in Cape Town and Johannesburg, South Africa. BMC Health Serv Res. 2021;21(1):17. doi:10.1186/s12913-020-05942-8 33407395 PMC7788832

[13] Roche SD Wairimu N Mogere P , et al. Acceptability and feasibility of pharmacy-based delivery of pre-exposure prophylaxis in Kenya: a qualitative study of client and provider perspectives. AIDS Behav. 2021;25(12):3871-3882. doi:10.1007/s10461-021-03229-5 33826022 PMC8602157

[14] Ortblad KF Mogere P Roche S , et al. Design of a care pathway for pharmacy-based PrEP delivery in Kenya: results from a collaborative stakeholder consultation. BMC Health Serv Res. 2020;20(1):1034. doi:10.1186/s12913-020-05898-9 33176785 PMC7661206

[15] Pintye J Odoyo J Nyerere B , et al. PrEP acceptability, initiation, and continuation within a commercial pharmacy-based PrEP delivery model for adolescent girls and young women in Kenya. Presented at IAS 2021 – the 11th IAS Conference on HIV Science; 2021; Virtual/Berlin, Germany. https://theprogramme.ias2021.org/Abstract/Abstract/160.

[16] Clarke V Braun V . Thematic analysis. J Posit Psychol. 2017;12(3):297-298. doi:10.1080/17439760.2016.1262613

[17] Nowell LS Norris JM White DE Moules NJ . Thematic analysis: striving to meet the trustworthiness criteria. Int J Qual Methods. 2017;16(1):1-13. doi:10.1177/1609406917733847

[18] Kruk ME Gage AD Arsenault C , et al. High-quality health systems in the sustainable development goals era: time for a revolution. Lancet Glob Health. 2018;6(11):e1196-e1252. doi:10.1016/S2214-109X(18)30386-3 PMC7734391 30196093

[19] Pintye J . Enhancing PrEP Outcomes among Kenyan Adolescent Girls and Young Women with a Novel Pharmacy-based PrEP Delivery Platform. 2022. 10.1186/s13063-024-08206-6 PMC11186170 38890744

[20] Donnell D Beesham I Welch JD , et al. Incorporating oral PrEP into standard prevention services for South African women: a nested interrupted time-series study. Lancet HIV. 2021;8(8):e495-e501. doi:10.1016/S2352-3018(21)00048-5 PMC8340029 34126052

[21] Hashim MJMD . Patient-centered communication: basic skills. Am Fam Physician. 2017;95(1):29-34. 28075109

[22] Rosenberg NE Bhushan NL Vansia D , et al. Comparing youth-friendly health services to the standard of care through “girl power-malawi”: a quasi-experimental cohort study. J Acquir Immune Defic Syndr. (1999). 2018;79(4):458-466. doi:10.1097/QAI.0000000000001830 PMC6203606 30085953

[23] Wilson KS Beima-Sofie KM Moraa H , et al. “At our age, we would like to do things the way we want”: a qualitative study of adolescent HIV testing services in Kenya. AIDS (London). 2017;31(Supplement 3):S213-S220. doi:10.1097/QAD.0000000000001513 PMC5497781 28665879

[24] Jonas K Duby Z Maruping K , et al. Perceptions of contraception services among recipients of a combination HIV-prevention interventions for adolescent girls and young women in South Africa: a qualitative study. Reprod Health. 2020;17(1):1-122. doi:10.1186/s12978-020-00970-3 32795366 PMC7427945

[25] Rousseau E Julies RF Madubela N Kassim S . Novel platforms for biomedical HIV prevention delivery to key populations — community mobile clinics, peer-supported, pharmacy-led PrEP delivery, and the use of telemedicine. Curr HIV/AIDS Rep. 2021;18:500-507. doi:10.1007/s11904-021-00578-7 34708316 PMC8549812

[26] Maloney K Ward A Krenz B , et al. Expanding access to parasite-based malaria diagnosis through retail drug shops in Tanzania: evidence from a randomized trial and implications for treatment. Malar J. 2017;16(1):6. doi:10.1186/s12936-016-1658-y 28049481 PMC5209819

[27] Rutta E Tarimo A Delmotte E , et al. Understanding private retail drug outlet dispenser knowledge and practices in tuberculosis care in Tanzania. Int J Tuberc Lung Dis. 2014;18(9):1108-1113. doi:10.5588/ijtld.14.0020 25189561

[28] Burson RC Buttenheim AM Armstrong A Feemster KA . Community pharmacies as sites of adult vaccination: a systematic review. Hum Vaccin Immunother. 2016;12(12):3146-3159. doi:10.1080/21645515.2016.1215393 27715409 PMC5215426

[29] Wilhelm M Bridgeman MB . Clinical updates on the rapid advancements in COVID-19 care: vaccine uptake and the retail pharmacist. Pharm Times. 2021;87(2):106.

[30] Le P-P Braunack-Mayer A . Perspectives on privacy in the pharmacy: the views of opioid substitution treatment clients. Res Social Admin Pharmacy. 2019;15(8):1021-1026. doi:10.1016/j.sapharm.2019.02.003 30777646

[31] Quilliam S . “I honestly didn’t know that I could”: the rise and rise of in-pharmacy sexual and reproductive health guidance. J Fam Plann Reprod Health Care. 2014;40(1):66-68. doi:10.1136/jfprhc-2013-100811 24347583

